# Geographic differences in the distribution of molecular subtypes of breast cancer in Brazil

**DOI:** 10.1186/1472-6874-14-102

**Published:** 2014-08-29

**Authors:** Filomena M Carvalho, Lívia M Bacchi, Kátia M Pincerato, Matt Van de Rijn, Carlos E Bacchi

**Affiliations:** 1 Department of Pathology, Faculdade de Medicina da Universidade de São Paulo, Av. Dr. Arnaldo, 455 – room 1149, São Paulo, SP 01246-903, Brazil; 2 Division of Pathology, Hospital das Clínicas, Universidade de São Paulo, São Paulo, SP, Brazil; 3 Department of Pathology, Stanford University Medical Center, Palo Alto, CA, USA; 4 Consultoria em Patologia, Botucatu, SP, Brazil

**Keywords:** Breast cancer, Epidemiology, Brazilian races, Intrinsic molecular subtypes

## Abstract

**Background:**

To compare the distribution of the intrinsic molecular subtypes of breast cancer based on immunohistochemical profile in the five major geographic regions of Brazil, a country of continental dimension, with a wide racial variation of people.

**Methods:**

The study was retrospective observational. We classified 5,687 invasive breast cancers by molecular subtype based on immunohistochemical expression of estrogen-receptor (ER), progesterone-receptor (PR), human epidermal growth factor receptor 2 (HER2), and Ki-67 proliferation index. Cases were classified as luminal A (ER and/or PR positive and HER2 negative, Ki-67 < 14%), luminal B (ER and/or PR positive, HER2 negative, and Ki-67 > 14%), triple-positive (ER and/or PR positive and HER2 positive), HER2-enriched (ER and PR negative, and HER2- positive), and triple-negative (TN) (ER negative, PR negative, and HER2- negative). Comparisons of the ages of patients and molecular subtypes between different geographic regions were performed.

**Results:**

South and Southeast regions with a higher percentage of European ancestry and higher socioeconomic status presented with the highest proportion of luminal tumors. The North region presented with more aggressive subtypes (HER2-enriched and triple-negative), while the Central-West region predominated triple-positive carcinomas. The Northeast—a region with a high African influence—presented intermediate frequency of the different molecular subtypes. The differences persisted in subgroups of patients under and over 50 years.

**Conclusions:**

The geographic regions differ according to the distribution of molecular subtypes of breast cancer. However, other differences, beside those related to African ancestry, such as socioeconomic, climatic, nutritional, and geographic, have to be considered to explain our results. The knowledge of the differences in breast cancer characteristics among the geographic regions may help to organize healthcare programs in large countries like Brazil with diverse economic and race composition among different geographic regions.

## Background

Breast cancer remains a major health problem responsible for 458,400 deaths worldwide in 2008 [1]. The molecular intrinsic subtypes discovered in 1999 provided additional information on the clinical outcome independent of conventional prognosticators such as tumor size, tumor grade and lymph node status [2]. More importantly, various molecular subtypes respond differently to various chemotherapy treatments, so an accurate subclassification is important for deciding treatment options [3-6].

Racial/ethnic differences have been observed among the different molecular subtypes [7-12] with the most documented being the relatively high incidence of basal type breast cancer (also called “triple negative”) among African-American women compared to Caucasian females [7,8,12,13]. Although non-white women, particularly of African descent, have a lower incidence of breast cancer, this particular race group present with more aggressive tumors and a higher mortality rate [13,14].

We investigated whether a similar effect of race could be present in Brazil, a country of a continental size with a wide variation in the distribution of people from various racial backgrounds in its five major geographic regions. Brazil is a large country with the fifth largest area and the fifth largest population in the world with a total of more than 190 million inhabitants according to the 2010 official census [15]. It has 26 states divided over five major geographic regions: North, Northeast, Central-West, Southeast and South. The social-economic situation and ethnic backgrounds vary greatly among these regions and include descendants from Amerindians, European immigrants, Asians, and Africans brought to the continent as slaves in the 18^th^ century. Although there is a tendency of one race predominant over another in these regions (for example, Amerindians in the North, blacks in the Northeast and Europeans in the South and Southeast), there are some ethnic variations in the Brazilian population mainly due to a high rate of interracial marriage. Anti-miscegenation and segregation laws have not been part of the Brazilian culture, so Brazil is a home to a population characterized by a color continuum between white and black races. This leads to a well-known difficulty in categorizing races because they lack a precise legal definition [16].

Previous studies actually demonstrated that in Brazil color, as determined by a physical evaluation, is a poor predictor of genetic ancestry estimated by molecular markers [17,18]. There remain, however, significant geographical differences in the racial makeup of the population, influenced by diverse geographic and economic characteristics of this country with the size comparable to a continent. The knowledge of possible differences in a large and ethnically complex country such as Brazil clearly would benefit not only the study of breast cancer in this country, but also the comprehension of the mechanisms involved in different molecular subtypes, not to mention the opportunity to develop more efficient strategies of prevention and early detection of breast cancer, particularly among the minorities.

In Brazil, federal law regulates mammographic screening and it has been offered to all women over 50 years of age since 2009. The number of new cases of breast cancer in Brazil estimated in 2014 was 57,120 [19]. Currently, there is no data regarding the distribution of breast cancer molecular subtypes in Brazil or even within individual states of this country. Our aim is to compare the distribution of the intrinsic molecular subtypes by the immunohistochemical surrogates in the five major geographic regions in Brazil.

## Methods

### Patient selection

All patient data were obtained from the files of Consultoria em Patologia, a private surgical pathology laboratory located in the city of Botucatu, State of São Paulo, Brazil. Consultoria em Patologia is a reference laboratory and analyzes over 5,000 breast cancer cases per year. These cases usually are sent by pathologists and oncologists from all five geographic regions of Brazil in order to be evaluated for predictive and prognostic immunohistochemical markers, i.e., estrogen and progesterone receptors (ER and PR), human epidermal growth factor receptor 2 (HER2) and Ki-67 proliferation index. Age at diagnosis and the state of origin of the patients were obtained from the pathological report. Geographic regions of Brazil were classified as North, Northeast, Central-West, South, and Southeast. We selected consecutive cases of invasive breast cancer from July 2009 to March 2011 with assessable immunohistochemical study of ER, PR, HER2 and Ki-67. One of the reasons this period of time was chosen is that all these cases had the immunohistochemistry study performed using exactly the same immunohistochemical protocol. Another reason was that we included only the cases sent for routine prognostic and predictive factors determination. We excluded in situ and microinvasive carcinomas as well the cases, which were sent for second opinion.

### Immunohistochemistry analysis

ER, PR, and HER2 status and Ki-67 proliferation index were determined at the time of the patient’s cancer assessment by an immunohistochemical method on a selected tumor block. ER and PR were considered positive with >1% of the nuclear staining in tumor cells, although in all cases except in three, ER and PR results showed > 10% of positive cells. HER2 was considered positive with a 3+ score and negative with a 0+ or 1+ immunoreactivity using the previous ASCO/CAP recommendation [20]. Also, based on ASCO/CAP recommendations, breast cancers with 2+ immunohistochemical scores were evaluated for fluorescence in situ hybridization (FISH), and a ratio of >2.2 of HER2 gene to chromosome 17 was considered positive for HER2 gene overexpression; ratio <1.8 was considered negative for HER2 amplification, and cases with a ratio between 1.8 and 2.2 were classified as equivocal for HER2 amplification [20]. The Ki-67 proliferation index was determined in the area with the highest Ki-67 nuclear labeling. A total of 300 proliferating and nonproliferating cells were counted, and the percentage of proliferating cells was calculated. Table 1 summarizes the specifications of the primary antibodies used.

**Table 1:** Source and dilutions of the antibodies and epitope retrieval methods used in this study

**Antibody to**	**Clone**	**Source**	**Dilution**	**Epitope retrieval**
**Estrogen receptor**	Rabbit monoclonal antibody, SP1	Thermo Scientific	1:500	Pressure cooker, 9 min
**Progesterone receptor**	Mouse monoclonal antibody, PgR636	Dako	1:1000	Pressure cooker, 9 min
**HER2**	Rabbit monoclonal antibody, SP3	Thermo Scientific	1:100	Microwave oven
**Ki-67**	Mouse monoclonal antibody, MIB1	Dako	1:600	Pressure cooker, 9 min

HER2: human epidermal growth factor receptor 2; Pressure cooker: citrate buffer (pH 6) (Tender Cooker, Nordic Wave, USA). Microwave: citrate buffer (pH 6), 15 min (Electrolux®, 900 W).

### Molecular subtype classification

Immunohistochemical surrogate markers (ER, PR, HER2, Ki-67, EGFR and CK5/6) were used to determine the molecular category of breast cancer as previously described in the following groups: luminal A (ER + and/or PR+, HER2- and Ki-67 < 14%), luminal B (ER + and/or PR+, HER2- and Ki-67 > 14%), triple-positive (TP) (ER + and/or PR+, HER2+), HER2-enriched (ER- and PR- and HER2+), and triple-negative (TN) (ER-, PR- and HER2-) [21,22]. HER2+ included all the cases with a score of 3+ by immunohistochemistry and cases with score 2+ but showing amplification demonstrated by FISH according to the guidelines of The American Society of Clinical Oncology (ASCO) and the College of American Pathologists (CAP) [20].

### Statistical analysis

Descriptive statistical analysis was used to characterize the distribution of the patient’s age at diagnosis, hormonal receptor status, HER2 status, and molecular subtypes for the total sample and by the geographic region. Comparisons of the age of patients between different geographic regions, molecular subtypes, ER/PR status, and HER2 status were performed using the Kruskal-Wallis test. Associations between molecular subtypes, hormonal receptor status, HER2 expression, and categories of patient’s age with geographic regions were tested by a chi-square test. Missing values were not included in our statistical analysis and were deleted list-wise. Statistical analyses were performed using MedCalc for Windows (version 11.5.0.0; MedCalc Software, Mariakerke, Belgium), and a *p*-value less than 0.05 was considered significant.

### Institutional approval

The study was approved by the Scientific Committee of the Department of Pathology of the Faculdade de Medicina da Universidade de Sao Paulo, and also by the Ethical Committee for Research Projects of the Hospital das Clinicas da Faculdade de Medicina da Universidade de Sao Paulo (CAPPesq) (protocol 311/10).

### STROBE statement

This study has adhered to the STROBE guidelines for observational studies.

## Results

**Figure 1:**
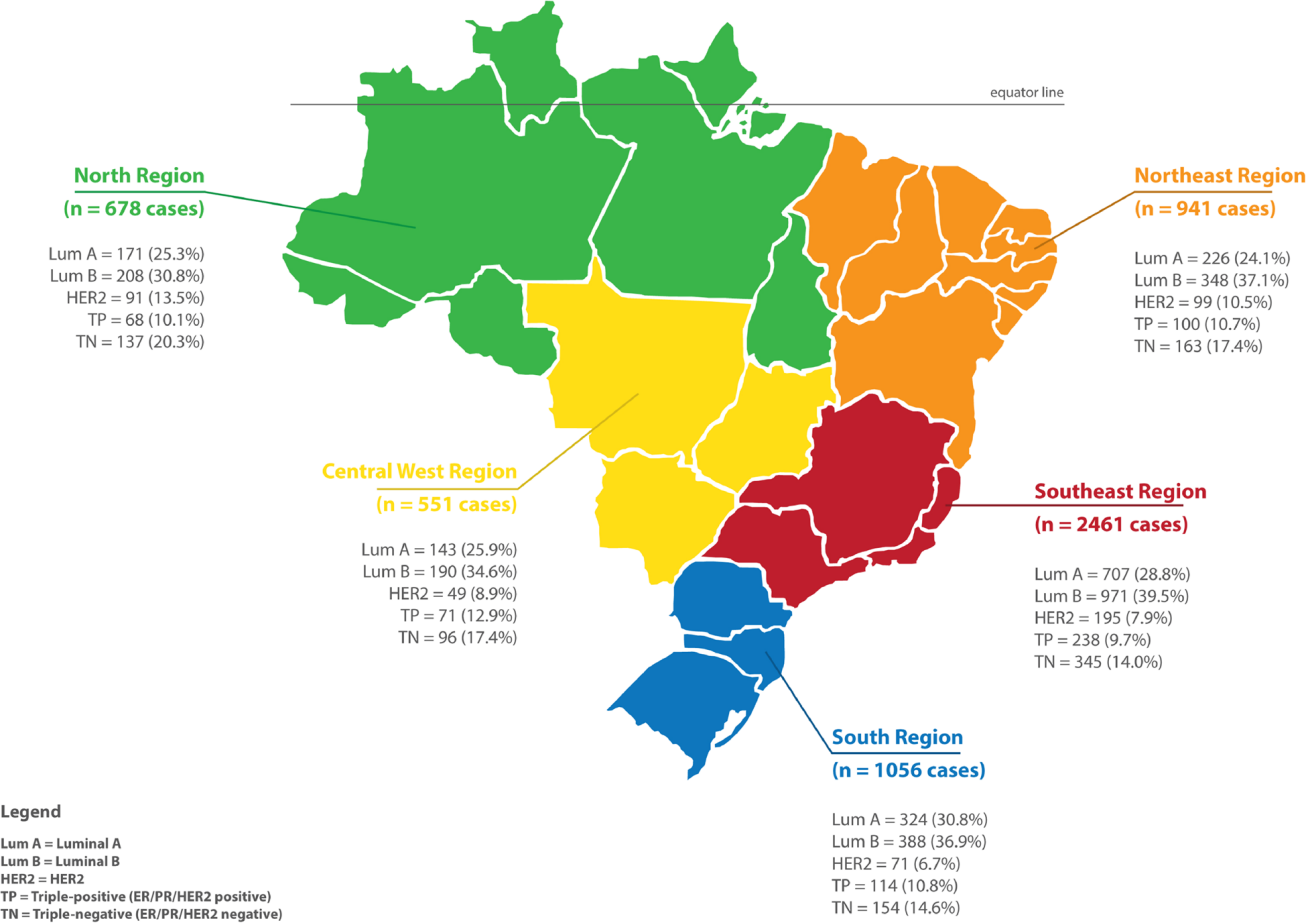
Brazilian map with the 5 geographic regions showing the distribution of 5,687 cases of breast cancer according to molecular subtypes determined by immunohistochemical surrogates.

In total, 5,687 eligible cases were included in the final analysis. The age of patients ranged from 16 to 98 years (mean 55.5 ± 13.5 years, median = 54 years). The distribution of age at diagnosis, hormonal and HER2 status, and the molecular subtypes are summarized in Table 2. In regional distribution, the age at diagnosis had the lowest mean in the North and Central-West regions and among patients with negative ER/PR, and HER2- positive tumors (Tables 2 and 3). Among molecular subtypes, however, the lowest mean age was seen among patients with a TP phenotype followed by TN carcinomas (Table 3). The distribution of the molecular subtypes differed in the five regions, which is illustrated in Figure 1. Luminal A carcinomas were more frequent in the Southeast (28.8%) and South (30.8%) regions. The highest proportion of luminal B carcinomas (39.5%) was seen in the Southeast region (*p* < 0.0001). Actually, the Southeast and South regions presented the highest proportion of ER/PR-positive tumors (*p* = 0.0003) (Table 2). HER2-enriched tumors were most frequent in the North region. The highest proportion of TP carcinomas came from the Central-West region. Triple negative carcinomas were more prevalent in the North region (*p* < 0.0001) (Table 2). The differences of frequency of the molecular subtypes in the five geographic regions persisted when we analyzed the subgroups of patients under 51 years (*p* = 0.0001) and over 50 years (*p* = 0.0012) (Table 4).

**Table 2:** Comparison of age of patients at breast cancer diagnosis, molecular subtype, expression of estrogen and progesterone receptor, and HER2 status among the five Brazilian geographic regions

**Variable**		**Brazilian geographic regions**					***p*-value**
		**SE**	**S**	**NE**	**CW**	**N**	
**Age**	(mean ± SD)	56.1 ± 13.5	55.8 ± 13.9	55.3 ± 13.7	54.3 ± 12.8	53.9 ± 13.1	<0.0001^a^
**Molecular subtype**	Luminal A	707 (28.8%)	324 (30.8%)	226 (24.1%)	143 (25.9%)	171 (25.3%)	<0.0001^b^
	Luminal B	971 (39.5%)	388 (36.9%)	348 (37.1%)	190 (34.6%)	208 (30.8%)	
	Triple-positive	238 (9.7%)	114 (10.8%)	100 (10.7%)	71 (12.9%)	68 (10.1%)	
	HER2	195 (7.9%)	71 (6.7%)	99 (10.5%)	49 (8.9%)	91 (13.5%)	
	Triple-negative	345 (14.0%)	154 (14.6%)	163 (17.4%)	96 (17.4%)	137 (20.3%)	
	Non-available^c^	4	5	3	2	3	
**ER/PR**	Positive	2039 (82.8%)	871 (82.5%)	753 (80.0%)	418 (75.9%)	529 (78.0%)	0.0003^b^
	Negative	422 (17.1%)	185 (17.5%)	188 (20.0%)	133 (24.1%)	149 (22.0%)	
**HER2**	Positive	433 (17.6%)	188 (17.8%)	201 (21.3%)	119 (21.6%)	163 (24.0%)	0.017^b^
	Negative	1972 (80.1%)	846 (80.1%)	725 (77.0%)	422 (76.6%)	506 (74.6%)	
	Score2+	56 (2.3%)	22 (2.1%)	15 (1.6%)	11 (2.0%)	9 (1.3%)	
**Total**		2461	1056	941	551	678	

^a^Kruskal-Wallis; ^b^chi-square; ^c^HER2 score 2+ and no FISH study to define the HER2 status.

**Table 3:** Comparison of age among different geographic regions, molecular subtypes, ER/PR status, and HER2 status

**Factor**		**n**	**Mean ± SD (years)**	**Significant difference (<0.05) from other factor(s)**^**a**^
**Geographic Region**	(1) CW	551	54.3	(5)
	(2) N	678	53.9	(3)(4)(5)
	(3) NE	941	55.3	(2)
	(4) S	1056	55.8	(2)
	(5) SE	2461	56.1	(1)(2)
**Molecular subtype**	(1) HER2	505	54.2 ± 12.8	(2)(5)
	(2) LUM A	1571	58.5 ± 13.0	(1)(3)(4)(5)
	(3) LUM B	2104	55.7 ± 13.7	(2)(4)(5)
	(4) TN	894	53.1 ± 13.4	(2)(3)(5)
	(5) TP	591	51.6 ± 13.0	(1)(2)(3)(4)
**ER/PR status**	(1) Positive	4610	55.6 ± 13.5	(2)
	(2) Negative	1077	53.4 ± 13.4	(1)
**HER2 status**	(1) Positive	1104	52.8 ± 12.9	(2)
	(2) Negative	4470	56.1 ± 13.6	(1)

^a^Kruskal-Wallis test, *p* < 0.0001 for all variables.

**Table 4:** Comparison of the molecular subtypes among the five Brazilian geographic regions according to age of patients at diagnosis

**Geographic regions**	**Molecular subtypes**									
	**Luminal A**		**Luminal B**		**Triple-positive**		**HER2**		**Triple-negative**	
**Age at diagnosis**	**<50y**	**≥50y**	**<50y**	**≥50y**	**<50y**	**≥50y**	**<50y**	**≥50y**	**<50y**	**≥50y**
**Southeast**	188/810 (23.2%)	519/1651 (31.4%)	332/810 (41.0%)	639/1651 (38.7%)	94 /810 (11.6%)	144/1651 (8.7%)	60/810 (7.4%)	135/1651 (8.2%)	135/810 (16.7%)	210/1651 (12.7%)
**South**	91/387 (23.5%)	233/669 (34.8%)	150/387 (38.7%)	238 /669 (35.6%)	57/387 (14.7%)	57/669 (8.5%)	25/387 (6.5%)	46/669 (6.9%)	63/387 (16.3%)	91/669 (13.6%)
**Northeast**	58/348 (16.7%)	168/592 (28.4%)	130/348 (37.3%)	217/592 (36.7%)	58/348 (16.7%)	42/592 (7.1%)	37/348 (10.6%)	62/592 (10.5%)	64/348 (18.4%)	99/592 (16.7%)
**Central West**	43/218 (19.7%)	100/332 (30.1%)	80/218 (36.7%)	110/332 (33.1%)	37/218 (17.0%)	34/332 (10.2%)	19/218 (8.7%)	30/332 (9.0%)	39/218 (17.9%)	56/332 (16.9%)
**North**	36/262 (13.7%)	135/415 (32.5%)	90 /262 (34.3%)	118/415 (28.4%)	28/262 (10.7%)	40/415 (9.6%)	35/262 (13.4%)	56/415 (13.5%)	72/262 (27.5%)	65/415 (15.6%)

Chi square test, patients <50y, *p* = 0.0001; Chi-square test, patients ≥50y, *p* = 0.001.

Table 5 summarizes the distribution of our cases, the official data of the Brazilian female population, and the incidence of breast cancer in the five geographical regions. Figure 1 illustrates the distribution of all the cases in this study according to the geographical regions.

**Table 5:** Distribution of 5,687 cases compared to overall and female Brazilian population among the five geographic regions, and incidence of breast cancer/100,000 women among females

**Geographic Regions**	**Total population**^**a**^	**Total female population**^**a**^	**Incidence of breast cancer/100,000**^**b**^	**Our cases**
**North**	15,864,454 (8%)	7,859,539 (8.1%)	21	678 (12%)
**Northeast**	53,081,950 (27%)	27,172,904 (27.9%)	37	941 (16%)
**Southeast**	80,364,410 (42%)	41,287,763 (42.4%)	71	2,461 (43%)
**South**	27,386,891 (14%)	13,950,480 (14.3%)	71	1,056 (18.6%)
**Central West**	14,058,094 (7%)	7,078,123 (7.2%)	51	551 (9%)
**Total**	190,755,799	97,348,809	56	5,687

^a^Data from Census 2010 IBGE [15]; ^b^Data from Brazilian National Cancer Institute (INCA) [19].

## Discussion

Brazil is a large country with a surface area of 8,511,960 km^2^ and with a population of 190,755,799. It is the most populous country in Latin America as well as one of the most populous in the world. The female population, according to the official 2010 census, is 97,348,809 [15]. We found significant differences in the proportion of the molecular subtypes of breast cancer between the five Brazilian geographic regions—each one with a distinct racial composition together with differences in climate, nutritional habits, urbanization and socioeconomic conditions.

The Brazilian racial composition is very heterogeneous with most populations descending from Europeans, Africans, Amerindians of various ethnic groups, and Asians. Most importantly, Brazil has experienced a high rate of intermarriage, and consequently, a mixed-race population has been created along five centuries of admixture.

A racial/ethnic influence on the presentation of breast cancer has been suggested in various studies [7,14,23-26] although race definition was neither very clear nor uniform in these studies. Most investigated race as a social characteristic without biologic basis of classification. Carey et al. conducted a population-based, case-controlled study with cases from the Carolina Breast Cancer Study and observed a higher prevalence of basal-like among premenopausal African Americans (39%) compared with postmenopausal African Americans (14%) and non-African American patients of any age (16%) [7]. Kurian et al. demonstrated different lifetime risks of the distinct subtypes defined by the ER, PR, and HER2 status among black, Hispanic, Asian, and white women [26]. The authors found a higher lifetime risk of TN tumors in black women and discussed the importance of this data on health policy and resource planning.

The high prevalence of basal-like tumors in premenopausal African Americans is pointed to as an important factor to the poorer prognosis in this group of patients [27,28], justifying the higher mortality rate despite the lower incidence [13,14]. Harper et al. documented 31/100,000 annual deaths among African Americans compared with 27/100,000 annual deaths for white women [29]. Delay in diagnosis due to disparities in healthcare access may contribute to higher rates of late-stage presentation and partially explain the increased rate of annual death in African Americans. In this study, we did not evaluate the stage or prognostic or outcome—factors influenced by social-economic status—but only the molecular subtype as a variable in geophysical distribution. In Brazil, besides the complexity of racial composition, the five geographic regions differ from each other regarding climate, urbanization, socioeconomic status, and nutritional habits— conditions that potentially can interfere in carcinogenesis as well as in the diagnosis and outcome of breast cancer.

In our study, Northern Brazil presented the highest proportion of TN carcinomas (20.3%). This region, largely covered by the Amazon rainforest, presents the largest Amerindian influences, both in culture and ethnicity. Indians represent 0.4% of the total Brazilian population [19], but they make up 1.5% of the Northern region. This region also has the highest African influence (77.8%), compared to 66.8% in Northeast, 65.9% in Central-West, 43.8% in Southeast, and 22.8% in South [15].

We must consider, however, the difficulty in defining race in Brazil and the need of a genomic classification. Pena et al. estimated the proportion of European, African, and Amerindian ancestry using a panel of 40 validated ancestry-informative insertion-deletion DNA polymorphisms in a population composed by black, white, and brown people from the four most populous Brazilian geographic regions (North, South, Northeast and South) [18]. The results were surprising as the authors were unable to demonstrate statistically significant differences among the four studied regions [18]. The authors found that European ancestry was predominant in all regions, varying from 60.6% in the Northeast to 77.7% in the South. Besides the difficulty to determine race in Brazil, these data show an important difference between the social and genomic determination of the African influence, particularly among the North population. The large area of Brazil has a variety of climate zones: equatorial, tropical, semi-arid, highland tropical and subtropical, each one with different life style, in part determined by the contribution of different ethnical groups, part by the substantial differences in the industrialization degree and social development.

The South region of Brazil houses the largest percentage of European descents, including German, Italian and Polish ancestry. White races comprise 80.6% of the population in this region compared to 24% in the North, 29.5% in the Northeast, 58.5% in the Southeast and 43.5% in the Central-West. According to our results, the South region showed the highest frequency of luminal A carcinomas—the more favorable molecular subtype—as well as a higher frequency of ER/PR-positive tumors, which is consistent with other studies [7,30,31]. According to the Brazilian National Institute of Cancer (INCA), Rio Grande do Sul, a state in the Southern region with a great influence of Italians and Germans boasts the highest incidence of breast cancer with 71 cases/100,000 women [19], which is consistent with other studies [14]. Our results indicated that most of our breast cancers located in this geographic region are a luminal A subtype and simultaneously presented with the lowest rate of HER2, TP, and TN tumors, suggesting an overall better prognosis.

The second region with a high proportion of luminal A and ER/PR-positive carcinomas was the Southeast, which was also the region with the second largest white population. The lowest incidence of luminal A was documented in the North, which presented a rate of breast cancer varying from 10 cases/100,000 women in the state of Acre to 26 cases/100,000 women in the state of Rondonia [19]. According to INCA, the only state of the North with a discrepant higher rate of breast cancer is Tocantins, which is located near the Central-West region, with 27 cases/100,000 women [19].

In addition to luminal A carcinomas, the luminal B category also was more prevalent in the South and Southeast and showed a lower frequency in the North region. It is important to stress that our luminal B did not include HER2-coexpression, like some authors have classified [7,11]. We analyzed the TP phenotype separately using classification criteria suggested by the previous St. Gallen consensus [32]. The TP phenotype was more frequent in the Central-West region, while HER2-enriched carcinomas were more prevalent in the North. In fact, our results indicated that more aggressive molecular subtypes are more prevalent in the North. This information must be considered in the planning of breast cancer prevention programs.

The distribution of HER2 carcinomas among different races/ethnic groups is more difficult to analyze because some authors included TP within the HER2 group [26], and others, within luminal B [7,11,13,30]. Fewer studies analyzed the group separately as we did. Kurian et al. did not find differences in the distribution of HER2- carcinomas, but they included TP among their HER2 tumors [26].

The differences in molecular subtypes that we observed among the five geographic regions persisted in the subgroups of patients under and over 50 years, suggesting the existence of a geographic phenomenon more than effect of age itself. Similar results have been reported by others [30]. Clarke et al. analyzed the distribution of the breast cancer subtypes among 91,908 patients from California and observed that black-women had higher rates of triple-negative carcinomas at all ages. The North region presented the lowest mean of age, and it was statistically different when compared to the Northeast, South, and Southeast.

Although we found significant differences in the distribution of molecular subtypes between the five geographic regions, we could not associate them to African ancestry, as other studies have been able to do in primarily the USA, despite the important African influence in the Brazilian racial composition. The reasons for this finding remain to be explored. One possible explanation is that the influence of African ancestry in Brazil may be different from that seen in the USA and Europe due to high rate of interracial marriage seen in Brazil. However, as we discussed above, there are other important factor to be considered,

There are studies that explored some genetic conditions implicated with racial/ethnic differences in breast cancer. Wang et al. observed a significantly higher methylation of the suppressor gene *CDH13* in breast tumor samples from African-American women when compared with European-American patients. The hypermethylation of the *CDH13* gene probably is related to early onset ER-negative breast cancer [33]. Several single-nucleotide polymorphisms (SNPs) have been associated to age and race of breast cancer patients [34]. Associations were observed for SNPs in *FGFR2*, *LSP1*, *H19*, *TLR1/TLR6* and *RELN* for African-Americans and in *FGFR2*, *TNRC9*, *H19* and *MAP3K1* for whites [34]. It is highly probable that the racial admixture can be responsible for some genomic differences, which are not necessarily similar to the ancestry.

Health disparities, either by racial/ethnic, socioeconomic, cultural, climatic, nutritional, or geographic are very complex to decode since there is a significant overlap between these factors [35]. Understanding the differences in breast cancer characteristics between geographic regions is the first step to organize healthcare programs. Moreover, this knowledge has important impact for the design and interpretation of clinical trials. As such, we believe that our study is relevant to determining the best strategy of health care and to better understand the tumor biology of breast cancer in large countries like Brazil with diverse economic and race composition among different geographic regions.

## Conclusions

The distribution of molecular subtypes of breast cancer differs by geographic region in Brazil, a country of continental dimension and a wide racial variation of people. These differences are multifactorial and must be taken into account for public health policy.

## Competing interests

The authors declare that they have no competing interests.

## Authors’ contributions

CEB: concept, performance of histological and immunohistochemical studies, and revision of the manuscript. LMB and KMP: collect of data and revision of the manuscript. MVR: concept and revision of manuscript. FMC: analysis and interpretation of data; writing of the manuscript. All authors read and approved the final manuscript.

## Pre-publication history

The pre-publication history for this paper can be accessed here:

http://www.biomedcentral.com/1472-6874/14/102/prepub

## References

[B1] Jemal A Bray F Center MM Ferlay J Ward E Forman D Global cancer statistics CA Cancer J Clin 2011 61 2 69 90 10.3322/caac.20107 21296855

[B2] Perou CM Sorlie T Eisen MB van de Rijn M Jeffrey SS Rees CA Pollack JR Ross DT Johnsen H Akslen LA Fluge O Pergamenschikov A Williams C Zhu SX Lønning PE Børresen-Dale AL Brown PO Botstein D Molecular portraits of human breast tumours Nature 2000 406 6797 747 752 10.1038/35021093 10963602

[B3] Caudle AS Yu TK Tucker SL Bedrosian I Litton JK Gonzalez-Angulo AM Hoffman K Meric-Bernstam F Hunt KK Buchholz TA Mittendorf EA Local-regional control according to surrogate markers of breast cancer subtypes and response to neoadjuvant chemotherapy in breast cancer patients undergoing breast conserving therapy Breast Cancer Res 2012 14 3 R83 10.1186/bcr3198 22621334 PMC3446346

[B4] Esserman LJ Berry DA Cheang MC Yau C Perou CM Carey L DeMichele A Gray JW Conway-Dorsey K Lenburg ME Buxton MB Davis SE van’t Veer LJ Hudis C Chin K Wolf D Krontiras H Montgomery L Tripathy D Lehman C Liu MC Olopade OI Rugo HS Carpenter JT Livasy C Dressler L Chhieng D Singh B Mies C Rabban J Chen YY Giri D Au A Hylton N I-SPY 1 TRIAL Investigators Chemotherapy response and recurrence-free survival in neoadjuvant breast cancer depends on biomarker profiles: results from the I-SPY 1 TRIAL (CALGB 150007/150012; ACRIN 6657) Breast Cancer Res Treat 2012 132 3 1049 1062 10.1007/s10549-011-1895-2 22198468 PMC3332388

[B5] Yunokawa M Koizumi F Kitamura Y Katanasaka Y Okamoto N Kodaira M Yonemori K Shimizu C Ando M Masutomi K Yoshida T Fujiwara Y Tamura K Efficacy of everolimus, a novel mTOR inhibitor, against basal-like triple-negative breast cancer cells Cancer Sci 2012 103 9 1665 1671 10.1111/j.1349-7006.2012.02359.x 22703543 PMC7659327

[B6] Martín M Rodríguez-Lescure A Ruiz A Alba E Calvo L Ruiz-Borrego M Santaballa A Rodríguez CA Crespo C Abad M Domínguez S Florián J Llorca C Méndez M Godes M Cubedo R Murias A Batista N García MJ Caballero R de Alava E Molecular predictors of efficacy of adjuvant weekly paclitaxel in early breast cancer Breast Cancer Res Treat 2010 123 1 149 157 10.1007/s10549-009-0663-z 20037779

[B7] Carey LA Perou CM Livasy CA Dressler LG Cowan D Conway K Karaca G Troester MA Tse CK Edmiston S Deming SL Geradts J Cheang MC Nielsen TO Moorman PG Earp HS Millikan RC Race, breast cancer subtypes, and survival in the Carolina Breast Cancer Study JAMA 2006 295 21 2492 2502 10.1001/jama.295.21.2492 16757721

[B8] Bauer KR Brown M Cress RD Parise CA Caggiano V Descriptive analysis of estrogen receptor (ER)-negative, progesterone receptor (PR)-negative, and HER2-negative invasive breast cancer, the so-called triple-negative phenotype: a population-based study from the California cancer Registry Cancer 2007 109 9 1721 1728 10.1002/cncr.22618 17387718

[B9] Yang XR Sherman ME Rimm DL Lissowska J Brinton LA Peplonska B Hewitt SM Anderson WF Szeszenia-Dabrowska N Bardin-Mikolajczak A Zatonski W Cartun R Mandich D Rymkiewicz G Ligaj M Lukaszek S Kordek R García-Closas M Differences in risk factors for breast cancer molecular subtypes in a population-based study Cancer Epidemiol Biomarkers Prev 2007 16 3 439 443 10.1158/1055-9965.EPI-06-0806 17372238

[B10] Kwan ML Kushi LH Weltzien E Maring B Kutner SE Fulton RS Lee MM Ambrosone CB Caan BJ Epidemiology of breast cancer subtypes in two prospective cohort studies of breast cancer survivors Breast Cancer Res 2009 11 3 R31 10.1186/bcr2261 19463150 PMC2716499

[B11] Chuang E Paul C Flam A McCarville K Forst M Shin S Vahdat L Swistel A Simmons R Osborne M Breast cancer subtypes in Asian-Americans differ according to Asian ethnic group J Immigr Minor Health 2012 14 5 754 758 10.1007/s10903-012-9577-7 22286607 PMC4051206

[B12] Ihemelandu CU Leffall LD Dewitty RL Naab TJ Mezghebe HM Makambi KH Adams-Campbell L Frederick WA Molecular breast cancer subtypes in premenopausal African-American women, tumor biologic factors and clinical outcome Ann Surg Oncol 2007 14 10 2994 3003 10.1245/s10434-007-9477-6 17647064

[B13] Ihemelandu CU Naab TJ Mezghebe HM Makambi KH Siram SM Leffall LD DeWitty RL Frederick WA Treatment and survival outcome for molecular breast cancer subtypes in black women Ann Surg 2008 247 3 463 469 10.1097/SLA.0b013e31815d744a 18376191

[B14] Chlebowski RT Chen Z Anderson GL Rohan T Aragaki A Lane D Dolan NC Paskett ED McTiernan A Hubbell FA Adams-Campbell LL Prentice R Ethnicity and breast cancer: factors influencing differences in incidence and outcome J Natl Cancer Inst 2005 97 6 439 448 10.1093/jnci/dji064 15770008

[B15] 2010 Population Census http://www.ibge.gov.br/english/estatistica/populacao/censo2010/default.shtm

[B16] Telles E Racial ambiguity among the Brazilian population Ethnic Racial Stud 2002 25 3 415 441 10.1080/01419870252932133

[B17] Parra FC Amado RC Lambertucci JR Rocha J Antunes CM Pena SD Color and genomic ancestry in Brazilians Proc Natl Acad Sci U S A 2003 100 1 177 182 10.1073/pnas.0126614100 12509516 PMC140919

[B18] Pena SD Di Pietro G Fuchshuber-Moraes M Genro JP Hutz MH Kehdy FS Kohlrausch F Magno LA Montenegro RC Moraes MO de Moraes ME de Moraes MR Ojopi EB Perini JA Racciopi C Ribeiro-Dos-Santos AK Rios-Santos F Romano-Silva MA Sortica VA Suarez-Kurtz G The genomic ancestry of individuals from different geographical regions of Brazil is more uniform than expected PLoS One 2011 6 2 e17063 10.1371/journal.pone.0017063 21359226 PMC3040205

[B19] Estimativa 2014. Incidência de Câncer no Brasil http://www.inca.gov.br/estimativa/2014/estimativa-24042014.pdf

[B20] Wolff AC Hammond ME Schwartz JN Hagerty KL Allred DC Cote RJ Dowsett M Fitzgibbons PL Hanna WM Langer A McShane LM Paik S Pegram MD Perez EA Press MF Rhodes A Sturgeon C Taube SE Tubbs R Vance GH van de Vijver M Wheeler TM Hayes DF American Society of Clinical Oncology; College of American Pathologists American Society of Clinical Oncology/College of American Pathologists guideline recommendations for human epidermal growth factor receptor 2 testing in breast cancer J Clin Oncol 2007 25 1 118 145 17159189 10.1200/JCO.2006.09.2775

[B21] Cheang MC Chia SK Voduc D Gao D Leung S Snider J Watson M Davies S Bernard PS Parker JS Perou CM Ellis MJ Nielsen TO Ki67 index, HER2 status, and prognosis of patients with luminal B breast cancer J Natl Cancer Inst 2009 101 10 736 750 10.1093/jnci/djp082 19436038 PMC2684553

[B22] Nielsen TO Hsu FD Jensen K Cheang M Karaca G Hu Z Hernandez-Boussard T Livasy C Cowan D Dressler L Akslen LA Ragaz J Gown AM Gilks CB van de Rijn M Perou CM Immunohistochemical and clinical characterization of the basal-like subtype of invasive breast carcinoma Clin Cancer Res 2004 10 16 5367 5374 10.1158/1078-0432.CCR-04-0220 15328174

[B23] Ihemelandu CU Naab TJ Mezghebe HM Makambi KH Siram SM Leffall LD Dewitty RL Frederick WA Basal cell-like (triple-negative) breast cancer, a predictor of distant metastasis in African American women Am J Surg 2008 195 2 153 158 10.1016/j.amjsurg.2007.09.033 18083134

[B24] Lantz PM Mujahid M Schwartz K Janz NK Fagerlin A Salem B Liu L Deapen D Katz SJ The influence of race, ethnicity, and individual socioeconomic factors on breast cancer stage at diagnosis Am J Public Health 2006 96 12 2173 2178 10.2105/AJPH.2005.072132 17077391 PMC1698157

[B25] Smigal C Jemal A Ward E Cokkinides V Smith R Howe HL Thun M Trends in breast cancer by race and ethnicity: update 2006 CA Cancer J Clin 2006 56 3 168 183 10.3322/canjclin.56.3.168 16737949

[B26] Kurian AW Fish K Shema SJ Clarke CA Lifetime risks of specific breast cancer subtypes among women in four racial/ethnic groups Breast Cancer Res 2010 12 6 R99 10.1186/bcr2780 21092082 PMC3046442

[B27] Ihemelandu CU Leffall LD Dewitty RL Naab TJ Mezghebe HM Makambi KH Adams-Campbell L Frederick WA Molecular breast cancer subtypes in premenopausal and postmenopausal African-American women: age-specific prevalence and survival J Surg Res 2007 143 1 109 118 10.1016/j.jss.2007.03.085 17950079

[B28] Dunn BK Agurs-Collins T Browne D Lubet R Johnson KA Health disparities in breast cancer: biology meets socioeconomic status Breast Cancer Res Treat 2010 121 2 281 292 10.1007/s10549-010-0827-x 20437200

[B29] Harper S Lynch J Meersman SC Breen N Davis WW Reichman MC Trends in area-socioeconomic and race-ethnic disparities in breast cancer incidence, stage at diagnosis, screening, mortality, and survival among women ages 50 years and over (1987-2005) Cancer Epidemiol Biomarkers Prev 2009 18 1 121 131 10.1158/1055-9965.EPI-08-0679 19124489

[B30] Clarke CA Keegan TH Yang J Press DJ Kurian AW Patel AH Lacey JV Age-specific incidence of breast cancer subtypes: understanding the black-white crossover J Natl Cancer Inst 2012 104 14 1094 1101 10.1093/jnci/djs264 22773826 PMC3640371

[B31] Cunningham JE Montero AJ Garrett-Mayer E Berkel HJ Ely B Racial differences in the incidence of breast cancer subtypes defined by combined histologic grade and hormone receptor status Cancer Causes Control 2010 21 3 399 409 10.1007/s10552-009-9472-2 20024610

[B32] Goldhirsch A Wood WC Coates AS Gelber RD Thürlimann B Senn HJ Members P Strategies for subtypes--dealing with the diversity of breast cancer: highlights of the St. Gallen International Expert Consensus on the Primary Therapy of Early Breast Cancer 2011 Ann Oncol 2011 22 8 1736 1747 10.1093/annonc/mdr304 21709140 PMC3144634

[B33] Wang S Dorsey TH Terunuma A Kittles RA Ambs S Kwabi-Addo B Relationship between tumor DNA methylation status and patient characteristics in African-American and European-American women with breast cancer PLoS One 2012 7 5 e37928 10.1371/journal.pone.0037928 22701537 PMC3365111

[B34] Barnholtz-Sloan JS Shetty PB Guan X Nyante SJ Luo J Brennan DJ Millikan RC FGFR2 and other loci identified in genome-wide association studies are associated with breast cancer in African-American and younger women Carcinogenesis 2010 31 8 1417 1423 10.1093/carcin/bgq128 20554749 PMC2950798

[B35] Januszewski A Tanna N Stebbing J Ethnic variation in breast cancer incidence and outcomes-the debate continues Br J Cancer 2014 110 1 4 6 10.1038/bjc.2013.775 24398563 PMC3887313

